# Community Preferences for the Allocation &Donation of Organs - The PAraDOx Study

**DOI:** 10.1186/1471-2458-11-386

**Published:** 2011-05-25

**Authors:** Kirsten Howard, Stephen Jan, John Rose, Steven Chadban, Richard DM Allen, Michelle Irving, Allison Tong, Germaine Wong, Jonathan C Craig, Alan Cass

**Affiliations:** 1 School of Public Health, University of Sydney, NSW, 2006, Australia; 2 The George Institute for Global Health, Camperdown, NSW, Australia; 3 Institute for Transport and Logistics Studies, University of Sydney, NSW, 2006, Australia; 4 Department of Renal Medicine, Royal Prince Alfred Hospital, Camperdown, NSW, 2050, Australia; 5 Sydney Medical School, University of Sydney, Sydney, Australia; 6 Director of Transplantation Services, Royal Prince Alfred Hospital, Camperdown, NSW, 2050, Australia; 7 Centre for Kidney Research, Westmead, NSW, Australia

## Abstract

**Background:**

Transplantation is the treatment of choice for people with severe organ failure. However, demand substantially exceeds supply of suitable organs; consequently many people wait months, or years to receive an organ. Reasons for the chronic shortage of deceased organ donations are unclear; there appears to be no lack of 'in principle' public support for organ donation.

**Methods/Design:**

The PAraDOx Study examines community preferences for organ donation policy in Australia. The aims are to 1) determine which factors influence decisions by individuals to offer their organs for donation and 2) determine the criteria by which the community deems the allocation of donor organs to be fair and equitable. Qualitative and quantitative methods will be used to assess community preferences for organ donation and allocation.

Focus group participants from the general community, aged between 18-80, will be purposively sampled to ensure a variety of cultural backgrounds and views on organ donation. Each focus group will include a ranking exercise using a modified nominal group technique. Focus groups of organ recipients, their families, and individuals on a transplant waiting list will also be conducted.

Using the qualitative work, a discrete choice study will be designed to quantitatively assess community preferences. Discrete choice methods are based on the premise that goods and services can be described in terms of a number of separate attributes. Respondents are presented with a series of choices where levels of attributes are varied, and a mathematical function is estimated to describe numerically the value respondents attach to different options. Two community surveys will be conducted in approximately 1000 respondents each to assess community preferences for organ donation and allocation. A mixed logit model will be used; model results will be expressed as parameter estimates (β) and the odds of choosing one option over an alternative. Trade-offs between attributes will also be calculated.

**Discussion:**

By providing a better understanding of current community preferences in relation to organ donation and allocation, the PAraDOx study will highlight options for firstly, increasing the rate of organ donation and secondly, allow for more transparent and equitable policies in relation to organ allocation.

## Background

There is an increasing recognition of the role and importance of community preferences in shaping public health policy. The field of organ donation and transplantation represents an area where the potential exists for policy to be made more effective and equitable through the inclusion of community preferences. The most pressing policy issue in this area is the chronic shortage of organs available for transplantation in Australia, as reflected in long waiting lists for transplant. A major factor contributing to this shortage in Australia is the low rate of organ donation.

In the PAraDOx study, we will examine two areas of organ transplant policy making. The first relates to preferences for organ donation - in particular, the factors that may influence decisions by members of the public to participate in organ donation after death. For instance some of the factors that may be considered are issues around consent, financial issues, the ability of donors to influence how their organs are allocated and the success rate of such transplants. The second relates to allocation of donor organs. Allocation in Australia, is carried out differently in different jurisdictions involving varying combinations of criteria including tissue matching, length of time on the waiting list and age. In the PAraDOx study, we will elicit community preferences for alternative allocation criteria with the aim of enabling policy to be set in line with community standards of fairness.

### Organ donation

Organ donation predominantly draws on deceased donors, although more than 40% of kidney donations in Australia are from living donors [1]. Live kidney donations are almost always made to known recipients, while organs from deceased donors are allocated to potential recipients on the transplant waiting list. In 2007, 1780 people were on transplant waiting lists around Australia, the vast majority waiting for kidney transplantation (1394) [2], with the average wait for a kidney from a deceased donor being about 4 years [3].

Australia has one of the lowest deceased organ donation rates in the developed world, with a donor rate of 13.8 donors per million population (dpmp)[2] compared with, for example, Spain (36.6 dpmp) and the United States (25.5 dpmp) [4]. Kidney transplantation, by far the most common form of organ transplant, is widely recognised as providing superior survival and quality of life over dialysis and significant cost savings to the community [5]; other organ transplants are life saving.

The reasons for Australia's low performance in deceased organ donation are unclear; there appears to be no lack of 'in principle' public support [3]. It is likely that both individual specific and systemic features may have an influence: individuals may register their willingness to donate their organs on the Australian Organ Donor Register, but ultimately the decision on whether to proceed to organ donation is made by the next of kin. A report from the Australian National Clinical Taskforce on Organ and Tissue Donation, established in 2006, highlighted the difficulties experienced by intensive care specialists in discussing organ donation with families [6]. Improving co-ordination and communication amongst health care providers may be one means for improving organ donor rates [3].

Spain with one of the highest organ donation rates in the world, operates a system of 'opting out', where consent is presumed unless stated otherwise. In the UK there have also been recent moves to adopt a 'presumed consent' system [7]. Despite overseas evidence of the effectiveness of such an approach [8], the effectiveness and ethical implications of such a system in Australia have not been explored.

Recent initiatives to bolster live donations of kidneys have also been proposed for Australia, including the reimbursement of costs to donors along the lines of programs in New Zealand [9] and Canada [10]. Significant financial and non-financial incentives have also been put forward as a means of encouraging donors [11-13]. At present, legislation across Australia prohibits any economic benefit to be furnished to donors reflecting laws in virtually every other country of the world except Iran [14]. Nevertheless a number of recent articles have re-opened the debate in this area, and it is clear that such payments, backed by effective regulation and harm minimisation measures, have strong supporters, particularly within the transplant and medical communities [11]. International surveys have indicated some support for broadening the measures available to policy makers to increase donor rates, including consideration of financial and non-financial incentives [13,15]. Ultimately the introduction of any such initiative would require first addressing the serious ethical concerns associated with payment for organs, and to do this we need to understand community preferences and attitudes surrounding this issue.

### Allocation of donor organs

The current shortage of donor organs highlights not only the need to explore innovative means of increasing supply but also the means by which existing organs are allocated. This requires assessment of the underlying principles that dictate firstly the criteria for including individuals on the transplant waiting list and then secondly, the basis for the allocation of available organs to those on the list[6].

There are differences in practices between Australian states in placing patients on waiting lists, particularly kidney transplant waiting lists [6,16]. There are also differences in waiting list placement based upon Indigenous status, with fewer than 5% of people aged under 65 years on the kidney transplant waiting list being Aboriginal/Torres Strait Islander patients [16]. These inequities are recognised by the Clinical Taskforce on Organ and Tissue Donation [6].

At present, allocation of deceased organs is based on national and state based algorithms determined largely by time on waiting list and tissue-matching. The relative weighting or importance of each of these criteria varies across state and national jurisdiction, and other criteria may also come into play. It is unclear how well these criteria accord with community values, and consequently there is an important role for a community surveys on these questions.

### Policy context

In recent years there has been a growing movement internationally toward the use of community consultation in health policy making including major initiatives in the UK and Canada [17-19]. In Australia, although there have been some attempts to explicitly use community preferences to develop health care policy through individual small-scale projects [20], the institutionalisation of such inputs into the policy making process has generally been limited.

Community consultation is seen to potentially play a pivotal role in organ donation and allocation health policy in Australia. The National Clinical Taskforce on Organ and Tissue Donation, established in October 2006, was charged with providing evidence-based advice on ways to improve the rate of safe, effective and ethical organ and tissue donation for transplantation in Australia. Six priority areas it considered essential to achieving national reform in the sector [6] have been indentified, including increasing community awareness and donor registration and improving organ allocation systems by adopting national organ allocation protocols across all states and territories to improve equity and transparency. Crucially, it is recognised that a better understanding of community attitudes and preferences for these issues is required "to ensure that clinicians and policy makers are in step with community values" [6].

The aims of the PAraDOx study are to

1) determine which factors influence decisions by individuals to offer their organs for donation and

2) determine the criteria by which the community deems the allocation of donor organs to be fair and equitable.

By providing a better understanding of current community preferences in relation to these issues, it is expected that the PAraDOx study will highlight options for firstly, increasing the rate of organ donation and secondly, allow for more transparent and equitable policies in relation to organ allocation.

## Methods/Design

### Overview of approach and methods

The PAraDOx study will utilise both qualitative and quantitative methods to explore community preferences and views on 1) organ donation and 2) organ allocation. Focus groups and one on one interviews will be conducted, and discrete choice methods will be used to quantitatively assess community preferences for organ donation and allocation.

### Discrete choice experiments (DCEs)

Discrete choice experiments involve surveys in which respondents are asked to choose between hypothetical alternatives defined by a set of differing attributes. This method is becoming more widely used in health as a means of quantifying patient and consumer preferences for health care policies and programs[21-24]. The method is based on the idea that goods and services, including health care services, can be described in terms of a number of separate attributes or factors. The levels of attributes are varied systematically in a series of questions and respondents choose the option that they prefer for each question. People are assumed to choose the option that is most preferred, or has the highest 'value'. From these choices, a mathematical function is estimated which describes numerically the value that respondents attach to different choice options. Other data collected in the survey, including attitudinal questions and sociodemographic information, may also enter the value functions as explanatory variables. Ultimately, DCE studies can determine which attributes are driving patient preferences, the trade-offs between attributes that people are willing to accept, and how changes in attributes can lead to changes in preferences and likely service uptake.

**Figure 1:**
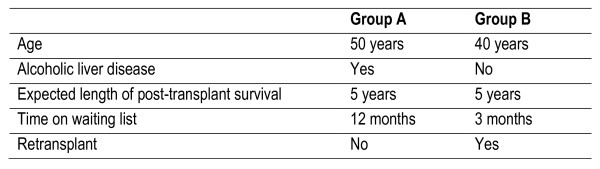
**Example of a discrete choice question**.

Figure 1 illustrates an example from Ratcliffe's [25,26] UK survey of community preferences for the allocation of donor liver grafts for transplantation. The example involves two unlabelled alternative groups of patients, Group A and Group B (Figure 1) described using five different attributes (age, whether patient had alcoholic liver disease, expected length of post-transplant survival, time on waiting list, whether it was a first or a re-transplant), each set at specific levels. By presenting respondents with a series of choices where the levels of the attributes are varied, researchers are able to quantify how these attributes influence choice. In this example, the analysis indicates community preferences for allocating livers to these patient groups based on the relative weight attached to each attribute.

Given a sufficient number of choices to allow variation across all attributes, this approach enables estimates of the marginal effect of each attribute on choice and the marginal rate of substitution or trade-offs between attributes. In principle this can be done by offering respondents choices using every combination of attributes; a 'full factorial' design. In practice such a design is rarely feasible; efficient designs are therefore paramount, particularly when considering multiple choice options and interactions between attributes and socio-demographic characteristics on choice.

### Study Methods

Two sub-studies will be conducted. The first examines community preferences for organ donation; the second will examine community preferences for organ allocation. The same methods and process will be utilised for each study, as described in the four steps below. The study will follow the ISPOR Guidelines for Good Research Practices for conjoint analysis in health[24].

### Stage 1: Focus group methods

Focus groups and one-on-one interviews will be conducted to establish the attributes to be included in the discrete choice experiments by determining the most important attributes and the range of feasible values within each. Focus groups will comprise 10-12 people, with a broad range of sociodemographic characteristics. Participants will be recruited via market research companies and will be grouped by age (18-25 years, 26-49 years and 50+ years) to facilitate communication with their peers. Participants will be purposively sampled to ensure a balance of numbers between male and female participants, a variety of cultural backgrounds and varying views on organ donation. Each focus group will have three phases; 1) preliminary questions about the participants' general thoughts and attitudes to organ donation, 2) a group discussion around factors that would influence participant's decisions to be an organ donor and 3) an individual ranking exercise of the factors identified from the group discussion. The ranking exercise will be conducted using a modified nominal group technique[27-29]. All sessions will be digitally audio-recorded and transcribed in full.

Additional focus groups of organ recipients and their families, and individuals on a transplant waiting list will also be conducted. Patients and families will be contacted through clinics in which the investigators are presently linked. The number of focus groups conducted will be based on when data saturation is achieved.

#### Organ donation

Focus group prompts will include 1) whether they would be an organ donor 2) whether there is anything that would make them not want to be an organ donor 3) their understanding of the consent process to become a donor 4) whether they would consider consenting to donating a family members organs 5) why they think Australia has such low donation rates compared to other western countries 6) what factors would make them (or others) more likely to donate their organs 7) what factors do they think are important to people when they make the decision to donate

#### Organ allocation

Focus group prompts will include: 1) how they think organs are allocated in Australia at the moment 2) what factors do they think should be considered in organ allocation 3) how should people be prioritised to receive organs. In addition, the focus groups will be augmented by one-on-one interviews with a convenience sample of physicians and surgeons involved in the transplantation process regarding their thoughts about the organ allocation policies and practices in Australia.

#### Focus group analysis

Transcripts will be entered into hyperRESEARCH (ResearchWare Inc. United States. Version 2.8.3), and reviewed line by line by the study team. A preliminary coding system will be developed using a grounded theory approach[30] The data from the focus groups and interviews will be analysed for emergent themes using qualitative methods of content and thematic analysis.

#### Nominal group analysis

Individual respondent rankings will be used to calculate importance scores for each factor identified in the focus groups. The highest ranked factor for each respondent will be given 20 points, the next most important given 19, and so on, progressively down to least important. Mean importance scores will be calculated. The percentage of respondents who ranked a factor in their top 10 will also be calculated. Differences across sociodemographic groups (for example age group, non-english speaking background, Australian state), and across respondent groups (for example community compared to patients), will be assessed using analysis of variance (ANOVA) for differences in mean importance scores of factors and χ^2 ^tests for differences in the proportions of respondents reporting factors in their top 10 rankings.

### Stage 2 Design of Discrete choice questionnaires

Once the attributes have been decided based on the qualitative work in Stage 1, a design for the discrete choice studies will be created. Statistically efficient designs will be used for both donation and allocation discrete choice studies. This approach to design links statistical efficiency to the likely econometric model that is to be estimated from choice data using the design [31,32]. This approach often lets go of the orthogonality constraint and attempts to minimise the expected asymptotic variance-covariance (AVC) matrix of the design. Efficient choice (EC) designs therefore attempt to maximise the likely asymptotic *t*-ratios obtained from choice data collected. As such, they attempt to minimize the correlation in the data for estimation purposes, and collect data such that parameter estimates have as small as possible standard errors. These designs make use of the fact that the AVC matrix (the roots of the diagonal of this matrix are the asymptotic standard errors) of the parameters can be derived if the parameters are known. Since the objective of the DCE is to estimate these parameters, they are unknown at the time of design. However, if some prior information about these parameters is available (e.g., parameter estimates available in the literature from similar studies, or parameter estimates from pilot studies), then this AVC matrix can be determined, assuming that the priors are correct.

Two initial EC designs will be created one for the organ donation study and one for the organ allocation study, based on the likely a priori sign of parameters. These initial designs will be piloted in a sample of 100 respondents for each study, and preliminary models estimated. Parameter estimates from the models will be used to generate the final efficient designs for the main discrete choice studies.

In addition to the discrete choice questions, information on socidoemographic characteristics of respondents will also be collected for each survey.

### Stage 3: DCE Survey

The DCE survey will be conducted using a web-based survey with quota sampling based upon age and sex to recruit a respondent sample broadly representative of the Australian public. Respondents will be recruited through a market research company with an existing online panel and experience in administering online choice based surveys. Upon consent, the potential respondent will be referred the online site to complete the discrete choice survey. Respondents will be asked to choose between different, unlabelled policy options which vary across a range of attributes.

#### Sample Size

The current theory of sampling for these experiments does not directly address the issue of minimum sample size requirements in terms of the reliability of the parameter estimates produced in the design of stated choice experiments (see for example, [33,34]). Rather, sampling theory as applied to choice modelling is designed to minimise the error in the choice proportions of the alternatives under study. This means that the final sample size required is based upon the characteristics of the design itself such as the number of attributes included, the attribute level range, the number of choice scenarios presented, the number of alternatives in each choice set and the size and direction of prior parameters obtained from the pilot study. To ensure that the population responses are broadly generalisable to the Australian adult population, and that we are able to explore interactions between attributes and between attributes and sociodemographic factors, and present subgroup analyses we anticipate a sample size of approximately 1000 respondents for each of the two surveys.

### Stage 4: Analysis

The results from this survey will inform policy by highlighting the factors that are likely to influence an individual's decision to become an organ donor and the factors that the community perceives as most important in the allocation of donor organs to those on transplant waiting lists.

A mixed multinomial logit (MMNL) (also known as random parameters logit, RPL) model using a panel size specification will be used for each analysis. A panel specification of the model allows for non-independence of observations provided by the same respondent; that is it can account for correlations amongst the multiple choices made by the same individual. MMNL models relax certain statistical assumptions of more commonly used multinomial logit (MNL) models, and often lead to models that better explain choice behaviour [33]. In MNL choice models, commonly used in health economics, parameters associated with each attribute are treated as fixed. These fixed values are the average (or point estimates) associated with a population level distribution; other information in the distribution is not considered. A MMNL allows consideration of the full distribution of a parameter estimate, and the fixed parameter becomes a random parameter. 'Random parameter' simply implies that each individual has an associated parameter estimate on that specified distribution. Whilst the exact location of each individual's preferences on the distribution may not be known, estimates of 'individual-specific preferences' can be accommodated by deriving the individual's conditional distribution, based - within sample - on their choices (i.e. prior knowledge) [35]. Interactions between attributes in the discrete choice surveys, and between attributes and population characteristics (for example, age, gender, income, education, knowledge of anyone who has received a transplant, non-english speaking background) will be explored in the mixed logit analysis for both studies.

Model results will expressed as parameter estimates (β), the odds of choosing one option instead of another (and 95% confidence intervals of the odds ratios) and p-values. Acceptable trade-offs between attributes will also be calculated.

### Ethical considerations

The PAraDOx study has been approved by the University of Sydney, Human Research Ethics Committee (Protocol numbers 12783, 12848 and 04-2011/13602), Sydney South West Area Health Service Ethics Review Committee (HREC/10/RPAH/23) and Sydney West Area Health Service Ethics Review Committee (SSA/10/WMEAD/92).

Confidentiality and anonymity of the data will be strictly maintained. Digital recording of the focus groups and physician interviews will only take place after written informed consent is obtained from participants. Participants will not be identifiable in any transcripts, or in any publications. It will be made clear to all participants that they have the right to withdraw from the research at any point in time.

As the population survey will be conducted as an online survey, written consent is not possible. As such participant information for the online survey will include the following statement "Being in this study is completely voluntary - you are not under any obligation to consent and - if you do consent - you can withdraw at any time without affecting your relationship with The University of Sydney. By completing the survey you have consented to be part of the study. You may stop completing the online survey at any point if you do not wish to continue, and we will not use your answers. Once you have submitted your survey anonymously, your responses cannot be withdrawn". As the survey is administered online, the study team will not have access to any information that could be used to identify respondents.

## Discussion

The PAraDOx study is a comprehensive analysis of community preferences for organ donation and organ allocation. Using qualitative and quantitative methods PAraDOx will provide an understanding of the views and preferences of the Australian community on alternative possible policy options for organ donation and allocation. Specifically, the aims of the PAraDOx study are to 1) determine which factors influence decisions by individuals to offer their organs for donation and 2) determine the criteria by which the community deems the allocation of donor organs to be fair and equitable.

The analysis will provide:

- Estimates of the marginal effect (importance) of each attribute on overall choice, e.g. if a payment attribute is presented in the study on organ donation, the analysis will provide an estimate of relative importance of receiving a payment on respondents' decision to donate.

- Estimates of marginal rates of substitution between attributes based on the ratio of parameter estimates, giving an indication of the extent to which respondents are prepared to trade-off one attribute for another. E.g. if payment and ability to influence organ recipient are offered as attributes in the survey, the marginal rate of substitution between these reflects the payment people are willing to accept as a trade-off for the ability to influence the recipient.

- An indication of the predicted uptake associated with different parameter levels within the estimated utility functions. This allows forecasting of, for instance, the level of organ donation uptake that could be expected given particular policy criteria (e.g. different types or levels of payment and ability to influence allocation) and socio-demographic characteristics.

By providing a better understanding of current community preferences in relation to these issues, the PAraDOx study will highlight options for firstly, increasing the rate of organ donation and secondly, allow for more transparent and equitable policies in relation to organ allocation.

## Competing interests

The authors declare that they have no competing interests.

## Authors' contributions

KH, SJ, AC were responsible for the conceptual design of the study. All authors participated in revisions to the study design and approved the final study design. KH, SJ were involved in drafting of the manuscript, other authors were involved in overall revision of the manuscript. All authors are involved in the implementation of the project, and have read and approved the final manuscript.

## Pre-publication history

The pre-publication history for this paper can be accessed here:

http://www.biomedcentral.com/1471-2458/11/386/prepub

## References

[B1] McDonald S Chang S Excell L ANZDATA Registry Report 2006 2006 Adelaide, South Australia: Australian and New Zealand Dialysis and Transplant Registry

[B2] Transplant Australia Statistics 2008 http://www.transplant.org.au/Statistic_s.html Internet (accessed May 2011)

[B3] Mathew T Faull R Snelling P The shortage of kidneys for transplantation in Australia Medical Journal of Australia 2005 182 5 204 5 15748126

[B4] Council of Europe Newsletter: Transplant 2006. International figures on organ donation and transplantation - 2005 2006 Spain: Council of Europe

[B5] Cass A Chadban SJ Craig JC The economic impact of end-stage kidney disease in Australia: Part I of the 'Study of the Economic Burden of Kidney and Urinary Tract Disease in Australia' 2006 Kidney Health Australia

[B6] National Clinical Taskforce on Organ and Tissue Donation Mid-Term Report for the Minister for Health and Ageing 2007 Canberra, ACT: Department of Health and Ageing

[B7] Hamm D Tizzard J Presumed consent for organ donation is an ethical and effective way of dealing with organ donation shortages BMJ 2008 336 230 10.1136/bmj.39475.498090.80 18244961 PMC2223059

[B8] Abadie A Gay S The impact of presumed consent legislation on cadaveric organ donation: A cross-country study Journal of Health Economics 2006 25 4 599 620 10.1016/j.jhealeco.2006.01.003 16490267

[B9] NZ Ministry of Social Development Financial assistance for live organ donors 2008 http://www.workandincome.govt.nz/documents/financial-assistance-for-live-organ-donors.pdf (accessed May 2011). Internet

[B10] The Kidney Foundation of Canada Living Organ Donor Expense Reimbursement Program Program FAQs 2006 http://www.transplant.bc.ca/FAQs.pdf (accessed May 2011). Internet

[B11] Friedman EA Friedman AL Payment for donor kidneys: pros and cons Kidney International 2006 69 6 960 2 10.1038/sj.ki.5000262 16482095

[B12] Jan S Thompson M Proposal: two part payment scheme for live kidney donors BMJ 2006 333 7561 26216873878

[B13] Kranenburg L Schram A Zuidema W Public Survey of Financial Incentives for Kidney Donation Nephrol Dial Transplant 2007 in press 10.1093/ndt/gfm643 18029378

[B14] Griffin A Kidneys on demand BMJ 2007 334 7592 502 5 10.1136/bmj.39141.493148.94 17347232 PMC1819484

[B15] Boulware LE Troll MU Wang NY Powe NR Public attitudes toward incentives for organ donation: a national study of different racial/ethnic and income groups American Journal of Transplantation 2006 6 11 2774 85 10.1111/j.1600-6143.2006.01532.x 16952292

[B16] Chadban S McDonald S Livingston B Excell L McDonald S, Excell L Transplant waiting list 2006 Adelaide, South Australia.: Australia and New Zealand Dialysis and Transplant Registry 103 24 ANZDATA Registry Report 2006 (To Dec 31 2005)

[B17] Davies C Barnett E Wetherall MS Citizens at the Centre. Deliberative Participation in Healthcare Decisions 2006 London: The Policy Press

[B18] McBride T Korczak V Community consultation and engagement in health care reform Australian Health Review 2007 31 Suppl 1 S13 S15 17402899 10.1071/ah070s13

[B19] Maxwell J Rosell S Forest P-G Giving citizens a voice in healthcare policy in Canada BMJ 2003 326 1031 3 10.1136/bmj.326.7397.1031 12742930 PMC1125934

[B20] Mooney GH Blackwell S Whose health service is it anyway? Community values in healthcare Med J Aust 2004 180 2 76 8 14723590 10.5694/j.1326-5377.2004.tb05804.x

[B21] Lancsar E Louviere J Conducting discrete choice experiments to inform healthcare decision making: a user's guide Pharmacoeconomics 2008 26 8 661 77 10.2165/00019053-200826080-00004 18620460

[B22] Bridges JF Kinter E Kidane L Things are looking up since we started listening to patients: Recent trends in the application of conjoint analysis in health 1970-2007 The Patient - Patient Centred Outcomes Research 2008 1 4 273 82 10.2165/1312067-200801040-00009 22272995

[B23] Marshall DA Bridges JF Hauber AB Conjoint Analysis Applications in Health - How are studies being designed and reported? An update on current practice in the published literature between 2005 and 2008 The Patient - Patient Centred Outcomes Research 2010 3 4 249 56 10.2165/11539650-000000000-00000 22273432

[B24] Bridges JF Hauber AB Marshall DA Conjoint Analysis Applications in Health--a Checklist: A Report of the ISPOR Good Research Practices for Conjoint Analysis Task Force. Value in Health 2011 14 4 5 10.1016/j.jval.2010.11.013 21669364

[B25] Ratcliffe J Buxton M Young T Longworth L Determining priority for liver transplantation: a comparison of cost per QALY and discrete choice experiment-generated public preferences Applied Health Economics & Health Policy 2005 4 4 249 55 10.2165/00148365-200504040-00007 16466276

[B26] Ratcliffe J Public preferences for the allocation of donor liver grafts for transplantation Health Economics 2000 9 2 137 48 10.1002/(SICI)1099-1050(200003)9:2<137::AID-HEC489>3.0.CO;2-1 10721015

[B27] Drennan V Walters K Lenihan P Priorities in identifying unmet need in older people attending general practice: a nominal group technique study Family Practice 2007 24 5 454 60 10.1093/fampra/cmm034 17675658

[B28] Corner J Wright D Hopkinson J The research priorities of patients attending UK cancer treatment centres: findings from a modified nominal group study British Journal of Cancer 2007 96 6 875 81 10.1038/sj.bjc.6603662 17342090 PMC2360101

[B29] Sanderson T Morris M Calnan M Patient perspective of measuring treatment efficacy: the rheumatoid arthritis patient priorities for pharmacologic interventions outcomes Arthritis care & research 2010 62 5 647 56 10.1002/acr.20151 20461786 PMC2886964

[B30] Charmaz K Constructing Grounded Theory - A practical guide through Qualitative anaylsis 2006 London: Sage Publications Ltd

[B31] Huber J Zwerina K The importance of utility balance in efficient choice design Journal of Marketing Research 1996 XXXIII 307 17

[B32] Sandor Z Wedel M Profile construction in experimental designs for mixed logit models Marketing Science 2002 21 4 455 75 10.1287/mksc.21.4.455.131

[B33] Hensher DA Rose JM Greene WH Applied Choice Analysis. A Primer 2005 1 Cambridge: Cambridge University Press

[B34] Louviere J Hensher DA Swait JD Stated Choice Methods - Analysis and Application 2000 Cambridge: Cambridge University Press

[B35] Hensher DA Greene WH Mixed logit models: state of practice Transportation 2003 30 2 133 76 10.1023/A:1022558715350

